# Different characteristics of mesenchymal stem cells isolated from different layers of full term placenta

**DOI:** 10.1371/journal.pone.0172642

**Published:** 2017-02-22

**Authors:** Yoo Shin Choi, Yong-Beom Park, Chul-Won Ha, Jin A Kim, Jin-Chul Heo, Woo-Jung Han, Soo-Young Oh, Suk-Joo Choi

**Affiliations:** 1 Department of Surgery, Chung-Ang University Hospital, Chung-Ang University College of Medicine, Seoul, South Korea; 2 Department of Orthopedic Surgery, Chung-Ang University Hospital, Chung-Ang University College of Medicine, Seoul, South Korea; 3 Department of Orthopedic Surgery, Samsung Medical Center, Sungkyunkwan University School of Medicine, Seoul, South Korea; 4 Stem Cell & Regenerative Medicine Institute, Samsung Medical Center, Seoul, South Korea; 5 Department of Health Sciences and Technology, SAIHST, Sungkyunkwan University, Seoul, South Korea; 6 Department of Obstetrics and Gynecology, Samsung Medical Center, Sungkyunkwan University School of Medicine, Seoul, South Korea; Montana State University Bozeman, UNITED STATES

## Abstract

**Background:**

The placenta is a very attractive source of mesenchymal stem cells (MSCs) for regenerative medicine due to readily availability, non-invasive acquisition, and avoidance of ethical issues. Isolating MSCs from parts of placenta tissue has obtained growing interest because they are assumed to exhibit different proliferation and differentiation potentials due to complex structures and functions of the placenta. The objective of this study was to isolate MSCs from different parts of the placenta and compare their characteristics.

**Methods:**

Placenta was divided into amniotic epithelium (AE), amniotic membrane (AM), chorionic membrane (CM), chorionic villi (CV), chorionic trophoblast without villi (CT-V), decidua (DC), and whole placenta (Pla). Cells isolated from each layer were subjected to analyses for their morphology, proliferation ability, surface markers, and multi-lineage differentiation potential. MSCs were isolated from all placental layers and their characteristics were compared.

**Findings:**

Surface antigen phenotype, morphology, and differentiation characteristics of cells from all layers indicated that they exhibited properties of MSCs. MSCs from different placental layers had different proliferation rates and differentiation potentials. MSCs from CM, CT-V, CV, and DC had better population doubling time and multi-lineage differentiation potentials compared to those from other layers.

**Conclusions:**

Our results indicate that MSCs with different characteristics can be isolated from all layers of term placenta. These finding suggest that it is necessary to appropriately select MSCs from different placental layers for successful and consistent outcomes in clinical applications.

## Introduction

Placenta is a very attractive source of mesenchymal stem cells (MSCs) as it is readily available and noninvasive without causing ethical issues [1,2]. In addition, maintenance of MSC stemness is excellent compared to that of the other tissues [3,4]. Several studies have reported that placental-derived MSCs have improved proliferative capacity, life span, and differentiation potential compared to bone marrow-derived MSCs [5–7]. Therefore, placental-derived MSCs are appropriate for clinical applications in terms of self-renewal and ease in isolation and acquisition with minimal ethical controversies.

The human placenta is a complex feto-maternal organ [8]. There has been a growing interest in isolating MSCs from parts of the placenta because it is assumed that they exhibit different *in vitro* proliferation and differentiation potential. Such differences appear to be caused by the complex structure and functions of the placenta. The placenta consists of amniotic epithelium (AE), amniotic membrane (AM), chorionic membrane (CM), chorionic trophoblast (CT), chorion villi (CV), and decidua (DC) [1,2,9]. Several studies have isolated and investigated the characteristics of AE [10–12], AM [13,14], CM [15], CT [16], CV [17], and DC [18,19]. However, no study has compared cells isolated from all layers of one full-term placenta. In addition, the feasibility of isolating MSCs from all placental layers is controversial. However, comparing MSCs from different parts of the placenta will facilitate the selection of appropriate MSCs for clinical applications.

In this study, we investigated the feasibility of isolating MSCs from different placental parts by dividing the placenta into AE, AM, CM, CV, chorionic trophoblast without villi (CT-V), and DC. The characteristics of the isolated MSCs were compared to those of MSCs isolated from whole placenta (Pla). We hypothesized that MSCs obtained from different layers of placenta would have different characteristics.

## Materials and methods

### Tissue processing

Full-term normal human placentas (n = 8) were collected from the Obstetrics Department at Samsung Medical Center. Written informed consent was obtained from the mothers. This study was approved by the institutional review board of Samsung Medical Center, Seoul, South Korea (SMC IRB File No.: 2006-02-034-001). Placenta-derived cells were prepared as follows. AE-derived MSCs were isolated from amniotic membrane layer as described previously [10]. Briefly, the amnion layer was mechanically peeled off from the chorion. The amniotic membrane (AM) includes two cells populations: mesenchymal and epithelial cells. The classic term “AM” referring to the membrane easily separated by peeling off from the chorionic membrane (CM) has been used in some previous studies [20,21]. This may cause confusion in differentiating specific tissue sources for MSC isolation. Therefore, we used term “gross AM” in this study. The “gross AM” is actually comprised of two different histologic layers: AE and “histologic AM”. Therefore, we used the term “AM” for “histologic AM (except AE)” in this paper regarding the specific source for cell isolation. The “gross AM” was washed 10 times with PBS without calcium or magnesium to remove blood. To release AE-derived MSC, “gross AM” was incubated at 37°C with 0.05% trypsin containing 0.5Mm EDTA (Invitrogen, Carlsbad, CA, USA). Cells obtained from the first 10 minutes of digestion were discarded to exclude debris. Cells from the second and third 30 minutes of digestion were pooled. Digested tissue sample was passed through a 70 μm cell strainer. The filtrate containing cell suspension was subjected to centrifugation at 1,200 rpm for 5 minutes. The cell pellet was re-suspended in low glucose DMEM (Gibco, Grand Island, NY, USA) supplemented with 10% fetal bovine serum (FBS) and 1% antibiotic-antimycotic (Gibco). Cells were then plated into100 mm^3^ dish. AE-derived MSCs were maintained at 37°C in a humidified atmosphere with 5% CO_2_ and allowed to attach for 72h. Non-adherent cells were removed. The culture medium was change twice a week. At 70~80% confluency, cells were harvested with TryPLE^™^ Express (Gibco, Grand Island, NY, USA) and plated into new dishes.

After separating the AE layer out as above, MSCs derived AM (“histologic AM”) were isolated as described previously [22]. Briefly, the AM tissue was minced and digested with 0.2% collagenase (Sigma, St. Louis, MO, USA) at 37°C for 1 h. Digested tissue sample was passed through a 70 μm cell strainer. The filtrate containing cell suspension was subjected to centrifugation at 1,200 rpm for 5 minutes. The cell pellet was re-suspended in low glucose DMEM supplemented with 10% FBS and 1% antibiotic-antimycotic. Cells were then plated into100 mm^3^ dish. AM-derived MSCs were maintained at 37°C in a humidified atmosphere with 5% CO_2_ and allowed to attach for 72h. Non-adherent cells were removed and culture medium was changed twice a week. At 70~80% confluency, cells were harvested with TryPLE^™^ Express and plated into new dishes.

The CMT layer was obtained by removing the amnion and decidua parietalis by careful scraping. The CM was then carefully separated from the CT using a #10 surgical blade. To isolate CM-derived MSCs, the CM layer was washed 10 times with PBS without calcium or magnesium to remove blood. It was then minced. To isolate CT-V-derived MSC, CT-V tissues were obtained by carefully scraping the soft trophoblastic tissue off the dense villi tissue using a #10 surgical blade. The obtained CT-V tissues were washed 10 times with PBS without calcium or magnesium to remove blood and then minced. The following procedures of digestion, filtration, re-suspension, and cultivation were the same as described above for AM tissue processing and for AM-derived MSC isolation. To isolate CV-derived MSC, CV tissues were obtained by carefully scraping the soft trophoblastic tissue out from the CT layer using a #10 surgical blade while leaving the dense villi tissue. The following procedures of mincing, digestion, filtration, re-suspension, and cultivation were the same as described above. Finally, DC derived MSCs were isolated as described previously [23]. Briefly, DC tissues were obtained by removing AM and CMT tissues by dissecting with sterile surgical scissors. The procedures used for isolating DC-derived MSCs were the same as described above.

MSCs isolated from a whole full-term Pla including the reflected membranes were used as control population. To obtain Pla-derived MSCs, whole placental tissue was washed 10 times with PBS without calcium or magnesium to remove blood and then minced. The same procedures as described above were then used afterward for each specific layer.

### Colony Forming Unit-Fibroblastic (CFU-F) assay

Cells (5 × 10^3^) were seeded onto 100 mm^2^ culture dishes, washed with PBS, and stained with 1% Giemsa solution (Sigma) in methanol after 10 days of culture. Cell clusters with diameter of 1–8 mm were counted as a colony under a microscope. The number of colonies was counted in five independent samples per experimental group.

### Growth kinetics study

Population doubling time (PDT) was estimated to compare the proliferation capacities of MSC populations. MSCs in passages 2–6 were counted for total cell number and cells (3 × 10^5^) were plated into 100 mm^2^ dishes. When cells reached 80–90% confluence, they were subcultured and the numbers were counted. Growth rate (GR), PDT, and cumulative population doubling (CDP) of MSCs were calculated for passages 2–6 with the following formulae: GR = (*N*_*f*_*—N*_*i*_)/(CT X 100cm^2^), PD = log(*N*_*f*_/ *N*_*i*_)/log2, and PDT = CT/PD (*N*_f_, final number of cells; *N*_i_, initial number of cells; PD, population doubling; and CT, culture time) [24]. Growth characteristics after long term culture and PDT were compared among MSC populations. The final passage of long-term culture was the passage in which cells did not proliferate twice or lose their fibroblastic shape.

### Fluorescence *in situ* hybridization

To determine the origin of MSCs isolated from different layers of placenta, we performed fluorescence in situ hybridization (FISH) analysis according to previously published procedures [25]. FISH analyses were performed for MSCs isolated from different layers of a placenta obtained from the donor who gave birth to a male child. All experiments were performed at passage 2. We used X and Y chromosome probes labeled with Cy3 (red) and fluorescein isothiocyanate (green), respectively, mapping to Xp11.1q11.1, a satellite centromeric region of the X chromosome, and Yq12, satellite III region of the Y chromosome (Vysis, Downers Grove, IL, USA). Slides were analyzed using an epifluorescence microscope with DAPI, spectrum red, and spectrum green filter under ×1000 magnification.

### Flow cytometry

Flow cytometry was performed for MSCs at passage 3. To determine cell surface marker expression, cell suspensions (5 × 10^5^ cells) were incubated with fluorescein isothiocyanate- or phycoerythrin-conjugated monoclonal antibodies specific for human mesenchymal and hematopoietic lineage markers at room temperature in the dark for 30 min. These antibodies were: CD44, CD73, CD90, CD105, CD31, CD34, CD 45, and HLA-DR (BD Bioscience, San Jose, CA, USA). Samples were sorted on a FACSAria flow cytometer. The resulting data were analyzed using CellQuest software (BD Bioscience).

### *In vitro* differentiation

Adipogenic, chodrogenic, and osteogenic induction were performed as described previously [26]. MSCs were cultured in adipogenic medium composed of DMEM plus 10% FBS containing 500 nM isobutylmethylxanthine (IBMX; Sigma), 20 μM indomethacin (Sigma), 10 nM dexamethasone (Sigma), and 10 μg/ml insulin (Sigma) for 3 days to induce adipogenic differentiation. They were then maintained in an adipogenic maintenance medium composed of DMEM plus 10% FBS containing 10 μg/ml insulin (Sigma) for 4 days. These procedures were repeated three times until the 21st day. Cultured cells were fixed and stained with Oil Red-O (Sigma) after 21 days of culturing. Lipid drop stains were solubilized with 100% isopropanol and the absorbance was measured at wavelength of 500 nm using a spectrometer.

To induce chondrogenic differentiation, cells (3 × 10^5^) were cultured in chondrogenic medium containing 10% FBS, 500 ng/mL bone morphogenetic protein-6 (R&D Systems, Minneapolis, MN, USA), 10 ng/mL transforming growth factor-β_3_ (Sigma), 0.1 μM dexamethasone (Sigma), 50 μg/mL ascorbic acid (Sigma), 40 μg/mL L-proline (Sigma), and 50 mg/mL insulin-transferrin-selenic acid premix (Sigma) in DMEM for 21 days. Cell pellets were harvested at 21 days post-induction and fixed in 4% paraformaldehyde overnight. Sections were prepared for Safranin O staining and collagen type II antibody treatment (Oncogene). Sulfated glycosaminoglycan (GAG) content was measured using a Glycan sulfated GAG assay kit (Biocolor Ltd., Carrickfergus, County Antrim, UK). These samples were digested with 0.2 M sodium phosphate buffer (pH 6.4) containing 125 μg/mL papain (Sigma), 10 mM L-cysteine hydrochloride (Sigma), and 2 mM EDTA (Sigma) at 65°C for 3 hours. The suspension was centrifuged at 10,000 × *g* for 10 min. The supernatant was mixed with 1 mL of Blyscan dye regent and shaken for 30 min. The precipitate was collected via centrifugation for 10 min and then dissolved in 500 μl dissociation reagent. Absorbance was measured in a 96-well plate at wavelength of 656 nm using a microplate reader.

MSCs were cultured under osteogenic culture conditions in medium containing 2 mM β-glycerophosphate, 100 μM L-ascorbic acid 2-phosphate, and 10 nM dexamethasone to induce osteogenic differentiation. After induction, cultures were stained with alkaline phosphatase on day 14 and with Alizarin Red on day 28. Cells were decalcified in 0.6 M HCl at 37°C for 24 hours to determine calcium accumulation. Calcium content was determined using the o-cresolphthalein complexone method according to the manufacturer’s instructions (Pointe Scientific, Canton, MI, USA). Briefly, acidic supernatant was reacted with o-cresolphthalein and the absorbance was measured at wavelength of 565 nm. Total calcium content was calculated from standard curves of known calcium concentrations. Calcium deposition values were normalized and quantified.

### Total RNA extraction and reverse transcription (RT)-PCR

Total RNA was extracted from MSCs using Trizol Reagent (Invitrogen) following the manufacturer’s instructions. Reverse transcribed (RT) reaction was performed at 42°C for 50 minutes using Superscript^™^ III reverse transcriptase (Invitrogen) and oligo-dT primer. The reaction was then incubated at 72°C for 15 minutes to inactivate the revesse transcriptase. For PCR reaction, the following specific oligonucleotide primers were used [27,28]: αP2-F (tactgggccaggaatttgac) and αP2-R (tcaatgcgaacttcagtcca) with PCR product of 240 bp in size; PPARγ2-F (ttcagaaatgccttgcagtg) and PPARγ2-R (tgggctccataaagtcaccaa) with PCR product of 599 bp in size; C/EBPα1-F (ctggagctgaccagtgacaa) and C/EBPα1-R (ccaagaattctcccctcctc) with PCR product size of 374 bp; Col1-F (tggagagtactggattgacc) and Col1- R (agtggtaggtgatgtttctgg) with PCR product size of 296 bp; ALP-F (acgtggctaagaatgtcatc) and ALP-R (ctggtaggcgatgtcctta) with PCR product size of 476 bp; Osteocalcin-F (gcgctacctgtatcaatg) and Osteocalcin-R (aggggaagaggaaagaag) with PCR product size of 315 bp; SOX9-F (ttgagccttaaaacggtgct) and SOX9-R (ctggtgttctgagaggcaca) with PCR product size of 631bp; Agreecan-F (tgagtcctcaagcctcctgt) and Agreecan-R (gtgccagatcatcaccacac) with PCR product size of 350 bp product; Col2A1-R (tcacgtacactgccctgaag) and Col2A1-F (aactgtgagagggtgggatg) with PCR product size 377 bp; Col10A1-F (tgggacccctcttgttagtg) and Col10A1-R (gccacacctggtcattttct) with PCR product size of 824 bp. These genes were used to determine adipogenic, osteogecin, and chondrogenic lineage differentiation. For the amplification of GAPDH (487bp product), primers GAPDH-F (gtcagtggtggacctgacct) and GAPDH-R (aggggtctacatggcaactg) were used. GAPDH was included as a positive control.

cDNA was used as template for PCR amplification using a Thermal cycler TP6000 (Takara Bio Inc co., Ltd). PCR conditions were: a initial denaturation at 95°C for 5 minutes followed by 30 cycles of 94°C for 30 seconds, specific annealing temperature for 30 seconds, and 72°C for 30 seconds. MSCs without induction were used as negative controls. PCR products were analyzed by electrophoresis using 1% agarose gel. GelRed^™^ (Biotium, Inc, Hayward, CA) was used in the gel for visualization under UV and photographic recording.

### Statistical analysis

Eight placenta samples were collected and processed to compare the characteristics of MSCs isolated from six different layers. Statistical analysis was done using Mann—Whitney test. MSCs obtained from Pla were regarded as reference for comparison. Statistical significance was considered at *P* < 0.05.

## Results

### Morphology and growth characteristics of cells isolated from different placental layers

**Fig 1:**
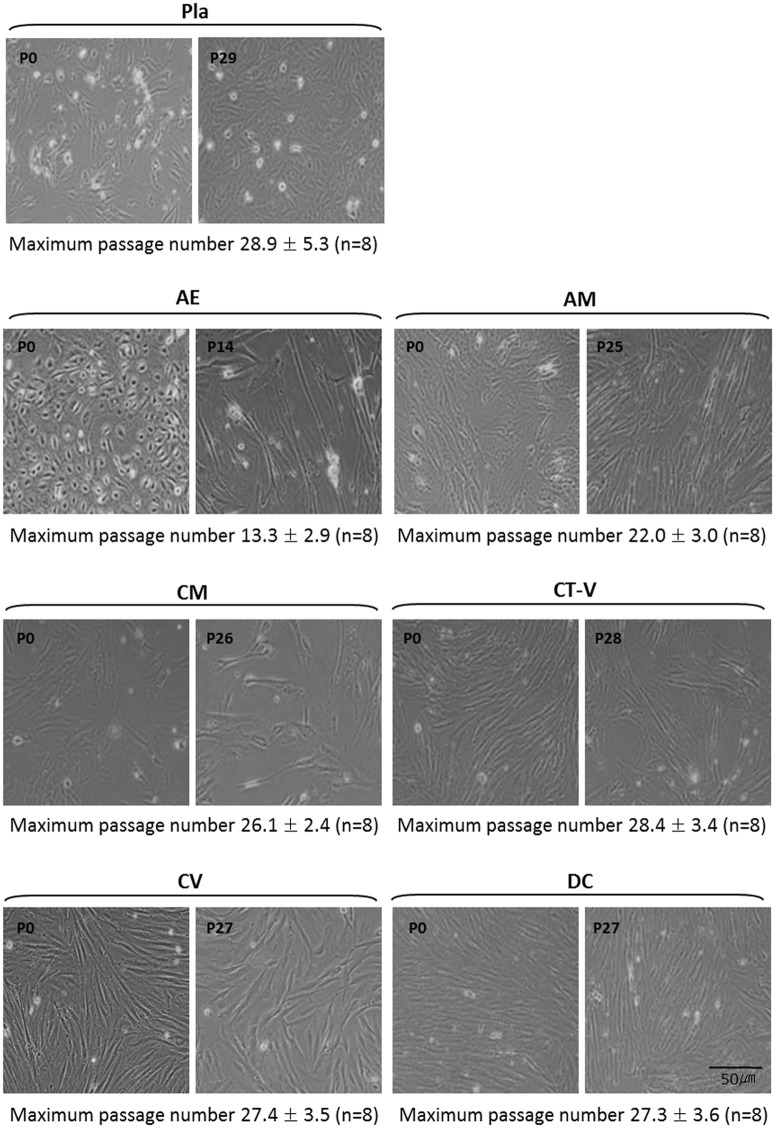
Culture of placental-derived stem cells from each layer at passage 0 and at the last passages. Scale bar: 50 μm. There were differences in the maximal number of passages in each specific layer where MSCs were derived. There was a tendency of longer maximum passages for mesenchymal stem cells isolated from the whole placenta (Pla-MSCs) but shorter maximum passages for mesenchymal stem cells isolated from AE among MSCs derived from specific layers of a single placenta. Pla-MSCs showed the largest variation in the maximum number of passages compared to MSCs from specific layers.

Regarding the morphology of cells isolated from each layer, cells isolated from AE were spheroidal at passage 0 and changed to a fibroblastic shape during passages 2 or 3. However, cells isolated from AM, CM, CT-V, CV, DC, and Pla were fibroblastic shape at passage 0 (Fig 1). Such fibroblastic shape was maintained thereafter (Fig 1). Culture was stopped when there was no doubling in cell numbers.

**Fig 2:**
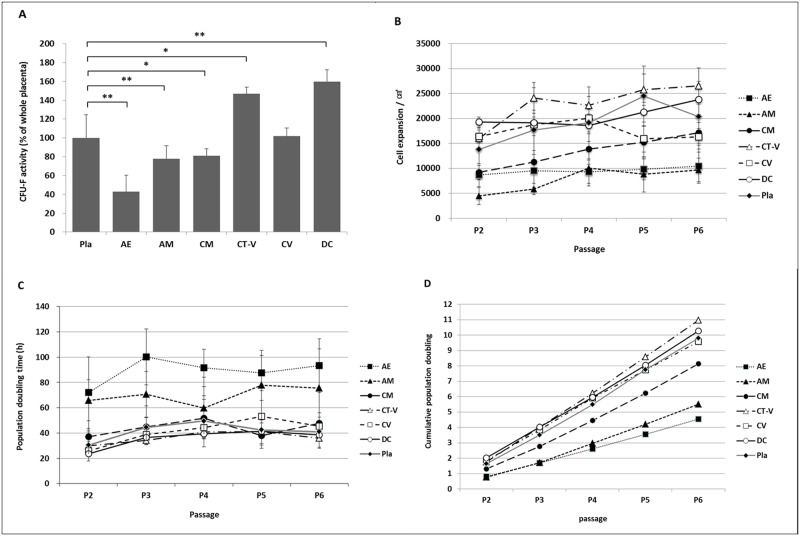
Proliferation of cells from different layers taken from a full-term placenta. (A) Relative quantification of CFU-F activity. (B) Growth rate from passages 2 to 6. (C) Proliferation doubling time from passage 2 to passage 6. (D) Cumulative population doubling. *, *P* < 0.05; **, P < 0.005.

CFU-F assay was performed at passage 2 to determine the self-renewal ability of these isolated cells. Results revealed that all cultures contained a subpopulation of cells capable of generating new fibroblast colonies from single cells. CFU-F activity differed among these different cell types. Cells from DC and CT-V showed higher activities compared to cells from Pla. CV cells showed similar activity to Pla cells. AE, AM, and CM cells had lower activities than Pla cells (Fig 2A). Cells derived from CT-V, CV, and DC showed higher growth rates compared to cells derived from AE, AM, and CM. CT-V cells showed the highest growth rate. Cell growth rates of CM, CT-V, and DC were increased from passage 2 to passage 6. However, cell growth rates of CV and Pla at passage 6 were decreased (Fig 2B).

PDT was evaluated at passages 2–6 to determine proliferation rates. The proliferative potentials of CM, CT-V, CV, and DC tended to be higher than those of AE and AM at all passages (Fig 2C). Mean PDT at passages 2–6 differed among Pla (41.57 ± 10.75 h), AE (88.92 ± 20.05 h), AM (69.94 ± 23.40 h), CM (43.82 ± 8.90 h), CT-V (36.42 ± 7.69 h), CV (41.52 ± 6.70 h), and DC (35.93 ± 7.52 h). AE and AM showed significantly higher PDT than Pla. CDP was increased from passage 2 to passage 6 in all histologic layers (Fig 2D). CDP in CT-V, CV, and DC was double compared to that of AE at passage 6.

These findings suggest that self-renewable cell populations with different proliferation rates in all layers of full-term placenta.

### Maternal and fetal contribution to Pla, AE, AM, CM, CT-V, CV, and DC populations

Results of FISH analysis revealed that MSCs isolated from AE, AM, CM, and CV were of fetal origin, whereas those from CT-V and DC were of maternal origin. Pla was of fetal or maternal origin. It was inconsistent depending on placental tissues obtained (S1 Fig).

### Characterization of MSCs isolated from all layers of full-term placenta

**Fig 3:**
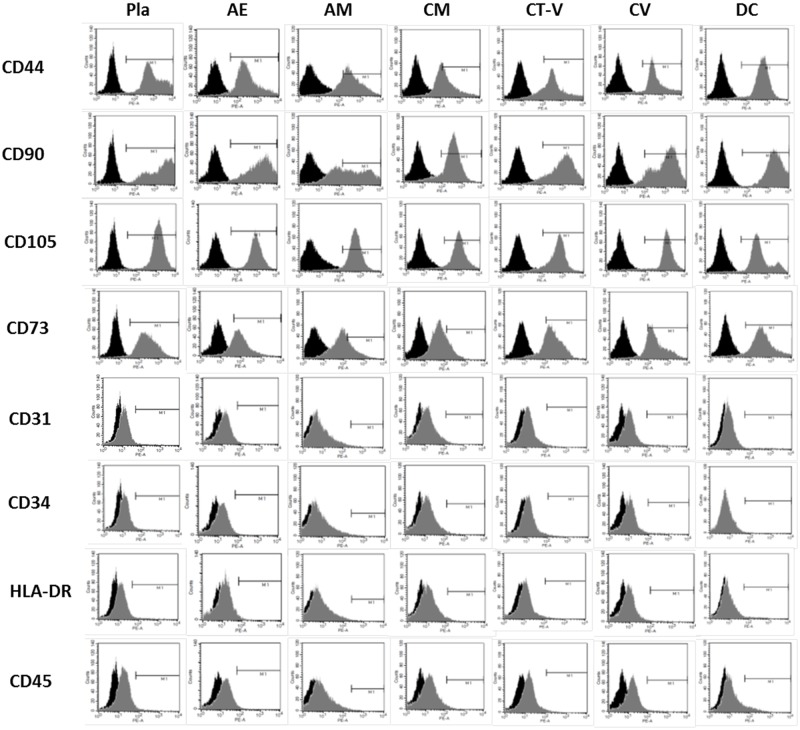
Flow cytometry analysis of cells from all layers to detect mesenchymal stem cell-specific antigens CD31, CD34, CD73, CD45, CD146, CD44, CD90, CD105, and HLA-DR during passage 3 compared to isotype controls (black).

Flow cytometry analysis was performed for passage 3 cultures containing cells isolated from all placental layers. Results of flow cytometry showed that cell surface markers (including the expression of CD44, CD73, CD90, and CD105) were highly expressed, consistent with the profiles of MSCs. All cells isolated from different placental layers were negative for CD34, CD45, and HLA-DR (Fig 3 and Table 1). These data indicate that cells isolated from all layers of full-term placenta have MSC characteristics.

**Table 1:** Surface marker expression in placenta-derived stem cells derived from each layer of placenta at passage 3.

	Pla (%)	AE (%)	AM (%)	CM (%)	CT-V (%)	CV (%)	DC (%)
**CD44**	98.62±1.22	98.92±2.26	95.02±5.45	95.33±3.88	98.22±1.37	98.57±2.70	98.22±1.69
**CD90**	97.48±3.28	98.10±1.81	96.87±3.69	98.68±1.09	95.50±5.44	98.88±1.35	98.08±1.26
**CD105**	99.45±0.80	99.57±0.54	99.52±0.46	95.87±1.84	99.37±0.29	99.70±0.41	97.80±3.25
**CD73**	92.23±4.49	90.45±3.60	89.18±4.59	91.25±2.14	90.57±5.42	94.53±4.21	90.22±7.08
**CD31**	0.47±0.33	0.08±0.13	0.08±0.20	0.20±0.17	0.27±0.18	0.18±0.22	0.17±0.14
**CD34**	0.35±0.32	0.20±0.14	0.17±0.19	0.25±0.25	0.12±0.15	0.23±0.19	0.22±0.08
**HLA-DR**	0.48±0.55	0.22±0.17	0.07±0.08	0.15±0.32	0.12±0.07	0.23±0.30	0.33±0.45
**CD45**	1.62±2.06	0.43±0.42	0.42±0.45	0.40±0.50	0.38±0.30	0.35±0.33	1.17±0.96

* Data are presented as mean ± standard deviation.

### Multi-lineage differentiation potential

**Fig 4:**
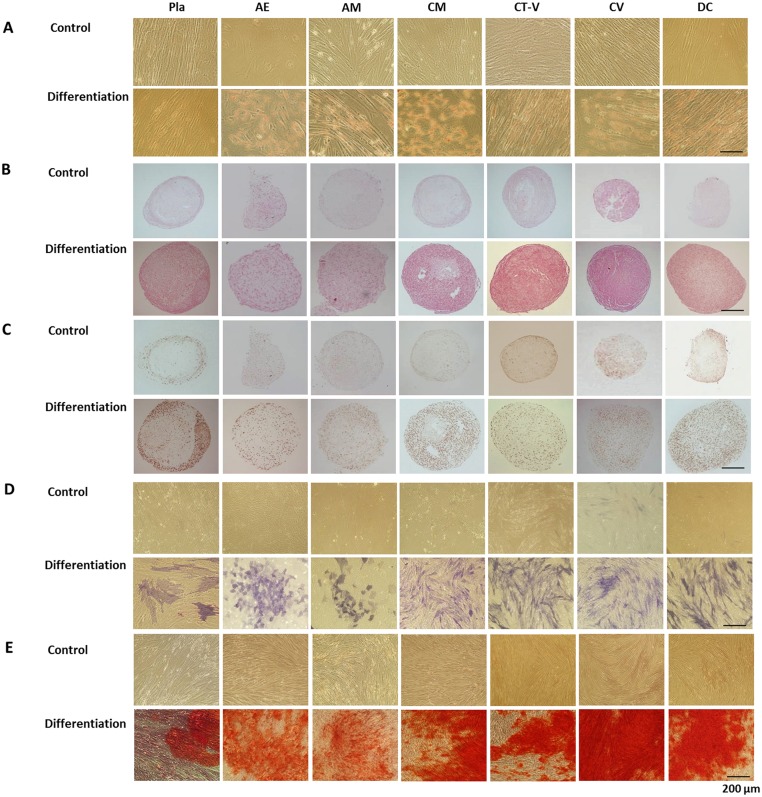
Multi-lineage differentiation of cells from different layers of a full-term placenta. Scale bar: 200 μm. (A) Adipogenic differentiation was confirmed by Oil Red-O staining of intracytoplasmic lipid droplets. (B) Chondrogenic differentiation of mesenchymal stem cells indicated by Safranin O staining. (C) Chondrogenic differentiation of mesenchymal stem cells indicated by positive type II collagen staining. (D) Osteogenic differentiation indicated by positive staining for alkaline phosphatase activity. (E) Osteogenic differentiation indicated by positive Alizarin Red-S staining.

**Fig 5:**
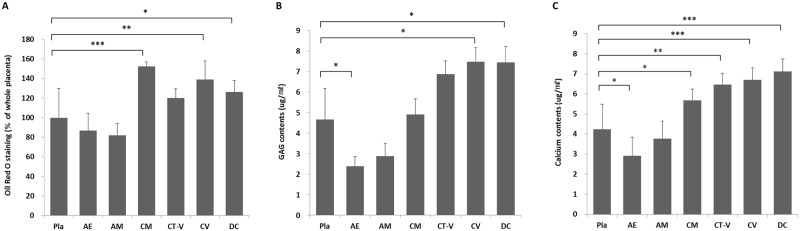
Relative quantification of cells from different layers of a full-term placenta to assess adipogenic (A), chondrogenic (B), and osteogenic (C) differentiation potentials. *, **, ***, *P* < 0.05, *P* < 0.005, and *P* < 0.001, respectively.

After 21 days of culture in adipogenic medium, cells were stained with Oil Red-O to detect the formation of lipid inclusions for adipogenic differentiation. All MSCs from different layers of the placenta showed adipogenic differentiation potentials (Fig 4A). The adipogenic differentiation potentials of MSCs from CM, CV, and DC layers were significantly higher than those of MSCs from Pla. MSCs from the CT-V layer tended to have high differentiation potential (*P* = 0.247, Fig 4A). MSCs from AE and AM layers tended to have lower differential potential compared to MSCs from Pla (AE vs. Pla, *P* = 0.123; AM vs. Pla, *P* = 0.123, Fig 5A).

After 21 days of culture in chondrogenic medium, cells were stained with Safranin O and collagen type II antibody to detect Safranin O-positive cell pellets for chondrogenic differentiation. All MSCs from different layers of the placenta showed chondrogenic differentiation potentials (Fig 4B and 4C). The amounts of GAG in MSCs derived from CV and DC layers were significant increased compared to those from Pla, indicating chondrogenic differentiation potential. However, MSCs derived from the AE layer showed significantly decreased chondrogenic potential compared to those derived from Pla (Fig 5B). MSCs from CM and CT-V layers tended to have high chondrogenic potentials than those from Pla (CM vs. Pla, *P* = 0.721; CT-V vs. Pla, *P* = 0.065, Fig 4B). MSCs from AM layer tended to have low chondrogenic potential compared to those from Pla (*P* = 0.083, Fig 5B).

Calcium mineralization in cells cultured in osteogenic medium was detected by alkaline phosphate and Alizarin Red-S staining to determine osteogenic differentiation. All MSCs from different layers of the placenta showed osteogenic differentiation potentials (Fig 4D and 4E). MSCs from CM, CT-V, CV, and DC layers showed significantly increased osteogenic differentiation potential compared to MSCs from Pla, whereas MSCs from the AE layer showed significantly decreased osteogenic differentiation potential compared to those from Pla (Fig 5C). MSCs from the AM layer tended to have lower osteogenic differential potential compared to those from Pla (*P* = 0.684, Fig 5C).

**Fig 6:**
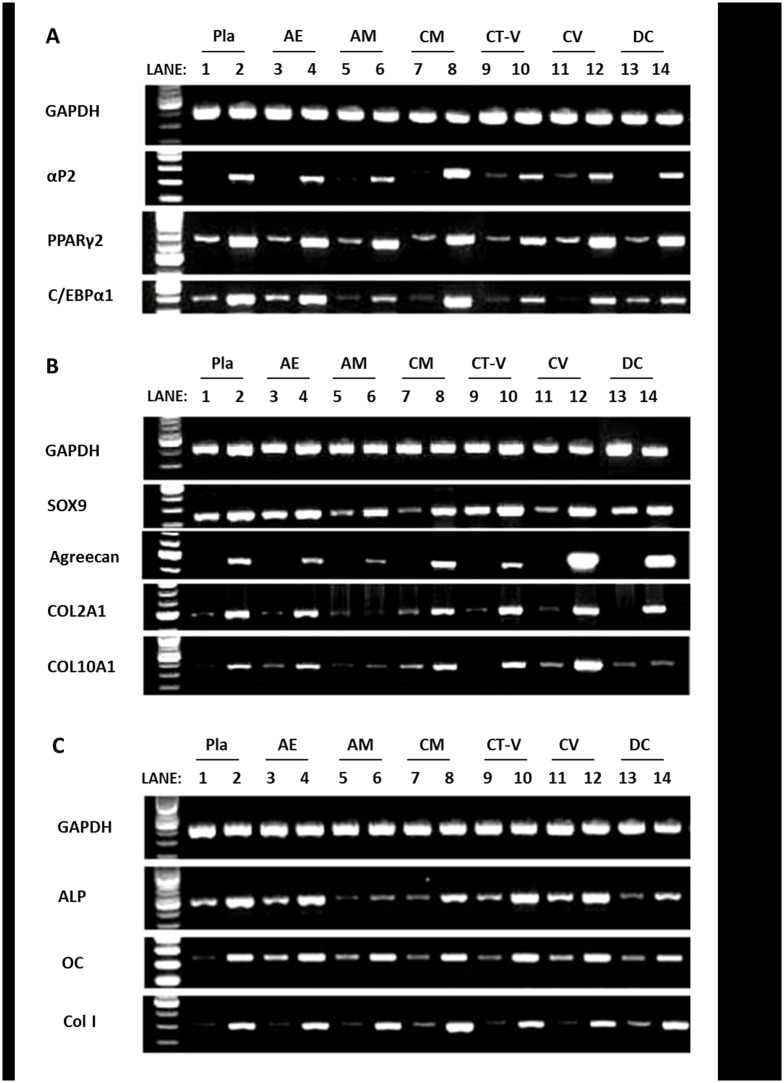
Gene expression based on RT-PCR analysis. (A) Adipogenic differentiation, (B) Chondrogenic differentiation, and (C) Osteogenic differentiation. Lanes 1,3,5,7,9,11 and 13: undifferentiated; Lanes 2,4,6,8,10 and 12: induced for differentiation. Housekeeping gene GAPDH was amplified to check the integrity of synthesized cDNA.

In RT-PCR after the induction of adipogenic, chondrogenic, or osteogenic differentiation, gene expression levels of adipogenic (aP2, C/EBPα 1, PPARγ2), chondrogenic (SOX9, Aggrecan, COL2A1, COL10A1), and osteogenic phenotype (ALP, osteocalcin, COL1A1) were increased in every MSC population from specific layers (AE, AM, CM, CT-V, CV, and DC) of the placenta. Especially, CM-derived MSCs showed high adipogenic gene expression, CV-derived MSCs showed high chondrogenic gene expression, while osteogenic gene expression was high in AE, CM, CT-V, CV, and DC-derived MSCs (Fig 6).

## Discussion

For the first time, this study showed that MSCs could be isolated from all layers of a full term placenta. We demonstrated that MSCs from various placental layers had different CFU-F, PDT, and multi-lineage differentiation potentials. We also provided evidence that MSCs from CM, CT-V, CV, and DC had more potential in terms of CFU-F, PDT, and multi-lineage differentiation potential. These findings indicate that MSCs from CM, CT-V, CV, and DC might have potential applications in regenerative medicine.

Our study demonstrated that MSCs could be isolated from all layers of full term placenta. Several *in vitro* studies have previously reported different characteristics of MSCs obtained from some portions of the placenta (by dividing the placenta into two or three portions) [29–31]. No previous studies, however, have isolated cells from each of the 6 layers of a single full term placenta and investigated the properties of these MSCs. To the best of our knowledge, this is the first report comparing MSC populations isolated from each of the 6 histologic layers of a single placenta. Previous studies have investigated cells from some layers of a full term placenta. In this study, we obtained populations of self-renewing fibroblast-like cells from all layers of a full term placenta. Immunophenotypic analysis results revealed that cells from all layers showed similar expression of surface markers with consistent MSC profile and strong expression of CD44, CD73, CD90, and CD105. However, CD34 and CD45 hematopoietic markers were not expressed. It has been reported that placenta-derived MSCs have constantly high proliferation rates up to passage 20. They can maintain their stemness after long-term culture [3]. All MSCs of the present study also maintained fibroblast-like shape after passage 20 except for cells derived from AE (passage 14). In addition, all MSCs showed multi-lineage differentiation potentials, confirming that placenta is an excellent source of adult MSCs.

In the present study, different characteristics of isolated MSC populations from each of the six histological layers were revealed. Using our method, a more homogenous MSC population could be obtained than a MSC population from a total placenta or a few divided portions of the placenta. MSCs obtained from various layers had different proliferation capacity and differentiation potential according to the source of the layer. Only one study has investigated the characteristics of cells from three layers (amniotic membrane, chorionic plate tissue, and decidua) of a term placenta in terms of surface antigen profile, proliferative capacity and differentiation ability [14]. In that study, MSCs from the amniotic membrane expressed high levels of a cell adhesion molecule surface marker compared to MSCs from the chorionic plate or the decidua. MSCs from the decidua had better growth characteristics and higher proliferation capacity compared to those from the amniotic membrane or chorionic plate [14]. In the present study, MSCs from CM, CT-V, CV, and DC showed higher proliferation capacities compared to those from AE and AM. The differentiation potentials of MSCs from CM, CT-V, CV, and DC were also higher compared to those from AE and AM. There were differences in the maximal number of passages, with a tendency of longer maximum passages for Pla-MSCs but shorter maximum passages for AE-MSCs. The added processing to separate specific layers might have affected the proliferation capacities of MSCs isolated from each specific layer. However, the largest variation in the maximum number of passages for Pla-MSCs seemed to be due to mixed presence of CT-V, CV, and DC MSCs in Pla-MSC population. These findings indicate that obtaining more homogeneous cell population is possible by isolating MSCs from a specific layer of placenta. We believe that the less variation seen in MSCs from specific layers should be considered as an advantage regarding future clinical application to obtain more consistent clinical outcome. Further investigations are needed to determine their clinical applications in regenerative medicine in the future.

Some limitations of this study need to be addressed. First, origin of cells from AE was not identified using epithelial markers (E-cadherin, Epcam). However, we used the same method to separate AE cells from the ‘gross AM layer (containing AE layer and histologic AM layer)’ as described in previous reports [32–34]. Therefore, we believe that AE cells could be isolated and obtained successfully from AE layer, separate from the ‘histologic AM layer’. In addition, the results of the present study showed that the characteristic of cells obtained from AE and ‘histologic AM’ were different. Moreover, the change of cellular morphology from epithelial to fibroblastic shape in AE cells observed in this study was similar to results of previous studies showing that epithelial cells obtained from the AE layer of placenta showed morphologic change to fibroblastic shape during culture expansion [35–37]. Therefore, we believe that cells could be isolated and obtained successfully from the AE layer, separate from the ‘histologic AM layer’. Second, Wharton’s jelly was not included in this study. Wharton’s jelly is a well-known specific source of MSCs. In the present study, our principal objective was to find a way to obtain more homogenous cell population from the complex placental tissue. Unfortunately, Wharton’s jelly was not included in our experiment. Third, repeated trypsin treatment might have affected the long term culture capacity and the maintenance of MSC-like properties. Previous studies of BM derived MSC subculture after repeated trypsin treatment have demonstrated a limitation of long term culture capacity (until passage 10) and the maintenance of MSC-like properties [38]. However, several studies have reportd the successful expansion of placenta-derived MSCs to more than 15–20 passages after repeated trypsin treatment while maintaining MSC-like properties [3,39,40]. Such difference in results could be due to several reasons. One possible reason is that the long term culture capacity with maintenance of MSC-like property is a characteristic feature of placenta tissue-derived MSCs. Another possibility is that our method of obtaining a more homogeneous cell population might have resulted in successful long term culture capacity. The fourth limitation of our study was that the maintenance of MSC characteristics and properties were only investigated between passages 2 to 6. Thus, we cannot tell whether these will be maintained after a long term culture. Considering the potential clinical application of MSCs, early passage MSC population is generally recommended, which was why we focused our study on the characterization of cells between passage 2 and 6. Since we could obtain sufficient number of cells from each layer in early passages, long term culture was unnecessary to obtain larger number of cells. We only performed long term culture to observe differences in long term culture capacity between MSC populations obtained from different layers. Fifth, the characteristics of differentiated MSC population and the capacity of ectodermal or endodermal lineage differentiation were not evaluated in this study. In the present study, we tried to reveal whether a more specific separation of the complex placental tissue could be of any benefit to obtain a more homogeneous MSC population considering their potential clinical applications [41,42]. Finally, placenta derived MSCs are known to have immunomodulatory properties. Unfortunately, we did not explore the immunomodulatory properties of MSC populations isolated from different layers of the placenta compared to MSCs derived from the whole placenta in this study. This could be a good topic for future investigations.

## Conclusions

We successfully isolated MSCs from all layers of a full term placenta. Surface antigen phenotype, morphology, and differentiation characteristics of these cells from all layers indicated that they exhibited properties of MSCs. Our results also demonstrated that MSCs from different layers of full term placenta had different proliferation rates and differentiation potentials. The results of this study suggest that obtaining MSCs from specific layers of placenta is possible. Careful selection of an appropriate layer is required for successful and consistent outcomes in clinical applications.

## Supporting information

S1 Fig Determination of fetal/maternal origin of isolated MSCs harboring an X (in red) and a Y (in green) chromosome after FISH (X1000). Analyses were performed for cells isolated from placental tissues obtained from a donor bearing a male child. Nuclei are labeled with DAPI (blue). (A) Fetal origin. (B) Maternal origin. Scale bar: 100 μm.
(DOCX)
