# Supplementary material for: Different characteristics of mesenchymal stem cells isolated from different layers of full term placenta
Source: PLoS One. 2017 Feb 22;12(2):e0172642. doi: 10.1371/journal.pone.0172642 (PMC5321410; doi:10.1371/journal.pone.0172642)
Supplement: S1 Fig — Analyses were performed for cells isolated from placental tissues obtained from a donor bearing a male child. Nuclei are labeled with DAPI (blue). (A) Fetal origin. (B) Maternal origin. Scale bar: 100 μm. (DOCX) [file pone.0172642.s001.docx]

**S1 Fig. Determination of fetal/maternal origin of isolated MSCs. harboring an X (in red) and a Y (in green) chromosome after FISH (X1000). Analyses were performed on the cells isolated from placental tissues obtained from donors bearing male children. Nuclei are labeled with DAPI (blue). (A) Fetal origin. (B) Maternal origin. Scale bar: 100 μm.**

**
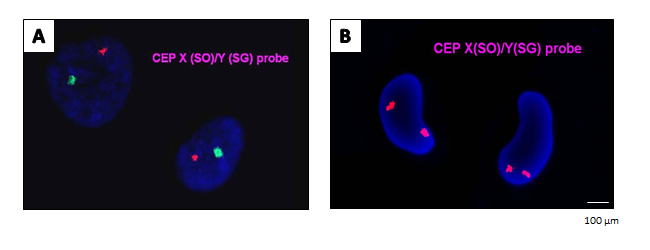
**
